# Boron Adsorption Using NMDG-Modified Polypropylene Melt-Blown Fibers Induced by Ultraviolet Grafting

**DOI:** 10.3390/polym15102252

**Published:** 2023-05-10

**Authors:** Ning Yu, Hui Jiang, Zhengwei Luo, Wenhua Geng, Jianliang Zhu

**Affiliations:** 1 College of Biotechnology and Pharmaceutical Engineering, Nanjing Tech University, Nanjing 211816, China; 202061218188@njtech.edu.cn (N.Y.); huijiang@njtech.edu.cn (H.J.); jlzhu@njtech.edu.cn (J.Z.); 2 School of Environmental Science and Engineering, Nanjing Tech University, Nanjing 211816, China; luozw2015@njtech.edu.cn

**Keywords:** ultraviolet grafting, PP melt-blown fiber, boron, adsorption, mechanism

## Abstract

Boron is in high demand in many sectors, yet there are significant flaws in current boron resource utilization. This study describes the synthesis of a boron adsorbent based on polypropylene (PP) melt-blown fiber using ultraviolet (UV)-induced grafting of Glycidyl methacrylate (GMA) onto PP melt-blown fiber, followed by an epoxy ring-opening reaction with *N*-methyl-*D*-glucosamine (NMDG). Using single-factor studies, grafting conditions such as the GMA concentration, benzophenone dose, and grafting duration were optimized. Fourier transform infrared spectroscopy (FT-IR), thermogravimetric analysis (TGA), scanning electron microscopy (SEM), X-ray diffraction (XRD), and water contact angle were used to characterize the produced adsorbent (PP-g-GMA-NMDG). The PP-g-GMA-NMDG adsorption process was examined by fitting the data with different adsorption settings and models. The results demonstrated that the adsorption process was compatible with the pseudo-second-order model and the Langmuir model; however, the internal diffusion model suggested that the process was impacted by both extra- and intra-membrane diffusion. According to thermodynamic simulations, the adsorption process was exothermic. At pH 6, the greatest saturation adsorption capacity to boron was 41.65 mg·g^−1^ for PP-g-GMA-NMDG. The PP-g-GMA-NMDG preparation process is a feasible and environmentally friendly route, and the prepared PP-g-GMA-NMDG has the advantages of high adsorption capacity, outstanding selectivity, good reproducibility, and easy recovery when compared to similar adsorbents, indicating that the reported adsorbent is promising for boron separation from water.

## 1. Introduction

Boron is a mineral that is frequently utilized in industry as a chemical raw ingredient. Dietary boron has an important role in embryonic development, bone metabolism, and immunological development in cattle and poultry breeding [1,2], and boron is a unique essential element of higher plants, among others. Boron is in high demand in industry, where it is used in glass, semiconductors, cosmetics, nuclear reactors, and radiation therapy [3,4]. China is a large industrial user of boron, importing large amounts of it every year due to limited mineral deposits and manufacturing capacity. Because China’s salt lake brine is rich in boron resources, the issue of boron extraction must be addressed immediately.

The precipitation technique [5,6], acidifying crystallization method [7,8], and membrane separation method [9] are the most frequently used procedures for boron extraction. Adsorption is one of the most successful technologies for boron removal, with the benefits of simplicity, environmental friendliness, and high efficiency [10]. *N*-methyl-*D*-glucosamine (NMDG) [11], o-binary phenols [12], and -hydroxyl carboxylic acids [13] are examples of boron adsorption functional groups. The borate anion group can bind with molecules having cis-ortho and inter-hydroxyl functional groups, resulting in a family of materials with boron affinity. Because boric acid and borate exist as B(OH)_4_^−^ in aqueous solutions, they may form stable chelation with neighboring hydroxyl groups [14].

Metal skeleton types [15,16], silicone [17], natural goods [18], and other commonly manufactured adsorbent material substrates are used. The majority of them are inorganic materials, while organic polymer materials as adsorbent substrate materials have received less attention. PP melt-blown fiber has excellent stability, high toughness, large specific surface area, large water flux, strong integrity, and simple recovery [19,20,21,22,23,24], and is stable for boron extraction in many situations. Nevertheless, because this polymer material is difficult to change [25,26], most studies have employed physical modification techniques to alter PP materials, with the two most popular techniques being plasma graft modification and ultraviolet (UV) graft modification. Luo Z W et al. [27] reported the preparation of surface ion imprinted polymers (IIP) using PP fibers as a substrate through plasma grafting technology to selectively adsorb hexavalent chromium. Haji A et al. [28] used oxygen plasma to modify the hydrophilicity of polypropylene nonwoven fabric followed by grafting *β*-cyclodextrin. Chan M A et al. [29] grafted polyethylene glycol diacrylate (PEGDA) onto microporous PP membranes by UV-initiated grafting to form moisture-sensitive porous structures. Sadeghi K et al. [30] used glycidyl methacrylate and 4-benzoyl phenyl methacrylate as raw materials to form a co-polymer coating on the surface of PP using the UV grafting technique. While both of these methods are good at modifying PP melt-blown fibers, UV grafting modification is cheaper and simpler compared to plasma grafting modification. This work uses UV grafting to modify PP melt-blown fiber to develop a boron-adsorbent material.

UV light grafting polymerization provides significant benefits over other surface modification processes for polymer materials. While long-wave UV light (300–400 nm) is not absorbed by polymer materials, it can be absorbed by a photo-initiator to start a reaction, allowing for surface modification without damaging the material body. The reaction can be carried out at room temperature, and the post-treatment is simple and free of contamination, making it a suitable approach for surface modification [29,31,32,33]. In this study, PP melt-blown fiber was grafted as intermediate glycidyl methacrylate (GMA) onto the surface of adsorbent with the UV grafting technique, then NMDG was attached to the surface of PP melt-blown fiber by the amination ring-opening process to create a boron adsorbent.

## 2. Experimental Part

### 2.1. Materials

Acetone, sodium hydroxide, boric acid, and hydrochloric acid were all obtained from Shanghai Lingfeng Chemical Reagent Co., Ltd. (Shanghai, China, AR). Ethanol was obtained from Shanghai Titan Technology Co., Ltd. (Shanghai, China, AR). GMA was obtained from Shanghai Aladdin Biochemical Technology Co., Ltd. (Shanghai, China, AR), Benzophenone (BP) from Shanghai Maclin Biochemical Technology Co., Ltd. (Shanghai, China, AR), and NMDG from Shanghai Yuanye Biotechnology Co., Ltd. (Shanghai, China, AR).

### 2.2. Preparation of Adsorbent

#### 2.2.1. PP Melt-Blown Fiber Pretreatment

Polypropylene melt-blown resin (Shanghai New Material Development Expert Co., Ltd., Shanghai, China) was used to create the PP melt-blown fiber. The PP melt-blown fiber was put in a conical flask, and an adequate quantity of acetone was poured to completely immerse it. Following 90 min of ultrasonic shaking, the PP melt-blown fiber was cleaned and dried in a vacuum drying oven for 2 h.

#### 2.2.2. PP-g-GMA Preparation

**Figure 1:**
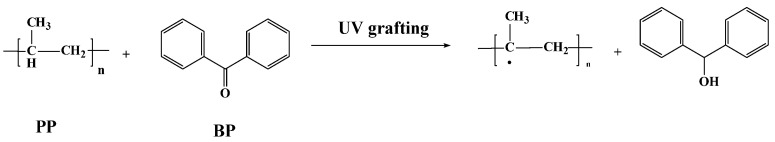
Principle of PP melt-blown fiber modified by UV grafting.

Tertiary hydrocarbon on PP melt-blown fiber is very unstable; while it can form tertiary carbon free radicals under the action of UV light, the amount of tertiary carbon free radicals spontaneously produced under the action of UV light is small, and cannot meet the requirements of grafting. Adding a photo-initiator can solve this problem [34]. BP was selected as the initiator in this experiment; the principle is shown in Figure 1.

To soak PP melt-blown fiber, a certain concentration of GMA (20%, 40%, 60%, 80%, 100%) and certain amount of BP (0.05 g, 0.10 g, 0.15 g, 0.20 g, 0.30 g) were combined, then the mixture was sonicated for 3 min and soaked for 15 min. The PP melt-blown fiber was poured into a plastic sealing bag after being removed. For exhaust, argon gas was supplied into the sealed bag. After exhausting, the sealed plastic bag was placed 10 cm under the UV light source (390 nm) and the PP melt-blown fiber was grafted for 2 min, 5 min, 8 min, 10 min, 13 min, and 15 min on both sides. After grafting, the grafted PP-g-GMA was rinsed with 50 cc of acetone in a round bottom flask. The refluxed PP-g-GMA was ultrasonically cleaned for 90 min before drying in a vacuum drying oven for 2 h. The UV grafting percentage (GP) of the PP-g-GMA was determined after drying, as follows:

(1) $$\begin{array}{c} \mathrm{GP}=\frac{m-m_{0}}{m_{0}}\times 100\% \end{array}$$

where *m*_0_ (g) represents the mass of PP melt-blown fiber before grafting and *m* (g) represents the mass of PP melt-blown fiber after grafting.

#### 2.2.3. PP-g-GMA-NMDG Preparation

**Figure 2:**
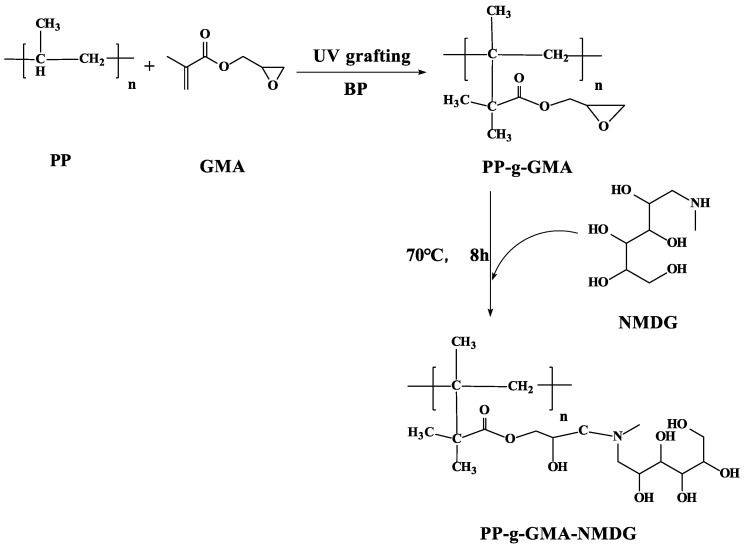
PP-g-GMA-NMDG synthesis roadmap.

A certain quantity of PP-g-GMA was dispersed in an aqueous 1,4-Dioxane solution (*v*/*v* = 4:1) and the same amount of NMDG was added to it. The mixture was heated to 70 °C by mechanical stirring, and the reaction lasted 8 h. The resulting PP-g-GMA-NMDG was washed with deionized water and ethanol before being vacuum dried for 2 h at 50 °C. Figure 2 depicts the preparation of PP-g-GMA-NMDG.

### 2.3. Characterization

The functional groups of the materials and intermediates were investigated using Fourier transform infrared spectroscopy (FT-IR, Nicolet 6700, Thermo Fisher, Waltham, MA, USA) in the 500–4000 cm^−1^ scanning range. An X-ray diffractometer (XRD, SmartLab, Tokyo, Japan) was used to measure X-ray diffraction patterns ranging from 10° to 90°. The adsorbent’s thermal stability was evaluated by thermogravimetric analysis (TGA, New Castle, DE, USA), with a maximum temperature of 800 °C in an N_2_ environment. SEM (Thermo Fisher, USA) was used to examine the surface morphologies of PP-g-GMA and PP-g-GMA-NMDG, and the contact angle (Theta Flex, Stockholm, Sweden) was used to evaluate the changes in hydrophilicity throughout manufacturing of the adsorbent.

### 2.4. Adsorption Experiments

The performance and mechanism of the PP-g-GMA-NMDG adsorbent were examined in terms of pH, temperature, time, solution concentration, adsorbent dose, adsorption selectivity, and reusability. Boron’s aqueous solution environment was simulated using boric acid aqueous solution. In the adsorption experiment, 10 mL of boric acid was utilized. In the single-factor experiment to evaluate grafting conditions, the boron concentration of the aqueous boric acid solution was 100 mg·L^−1^, the adsorbent dose was 1.0 g·L^−1^, and the adsorption volume was 10 mL. In this experiment, the concentration of all boron elements was determined by ICP-OES (iCAP 6300, Thermo Fisher, USA), and the adsorption quantity was computed per boron element. The following equation was used to compute the adsorption volume at adsorption equilibrium:

(2) $$\begin{array}{c} q_{e}=\frac{\left(C_{0}-C_{e}\right)V}{M} \end{array}$$

where *q*_e_ (mg·g^−1^) is the adsorption amount at adsorption equilibrium, *C*_e_ (mg·L^−1^) is the adsorption liquid’s equilibrium concentration, *C*_0_ (mg·L^−1^) is the adsorption liquid’s starting concentration, *V* (L) is the adsorption volume, and *M* (g) is the adsorbent’s mass.

#### 2.4.1. pH

The boron content in boric acid aqueous solution was set at 200 mg·L^−1^, and the pH was changed to 2, 4, 6, 8, and 10 with 1.0 mol·L^−1^ hydrochloric acid and sodium hydroxide, respectively. In a strain bottle, 1.0 g·L^−1^ of PP-g-GMA-NMDG and boric acid solutions of various pH values were combined and the constant temperature oscillation chamber was adjusted to 25 °C and 200 r·min^−1^ for 2 h. The correlation between solution pH and adsorption capacity was investigated.

#### 2.4.2. Adsorption Isotherm

Boric acid solutions with varying boron concentrations (50–600 mg·L^−1^) were produced, and PP-g-GMA-NMDG was added at a concentration of 1.0 g·L^−1^. PP-g-GMA-NMDG and boric acid solutions with varying boron concentrations were placed in strain bottles and shaken for 2 h at 25 °C and 200 r·min^−1^. Fitting was performed using the Langmuir and Freundlich models [35].

The Langmuir equation is represented as

(3) $$\begin{array}{c} q_{e}=k_{L}q_{m}C_{e}\left(1+k_{L}C_{e}\right) \end{array}$$

Its linear formulation is as follows:

(4) $$\begin{array}{c} \frac{C_{e}}{q_{e}}=\frac{C_{e}}{q_{m}}+\frac{1}{k_{L}q_{m}} \end{array}$$

where *C*_e_ (mg·L^−1^) represents the equilibrium concentration, *q*_e_ (mg·g^−1^) represents the equilibrium adsorption capacity, *q*_m_ (mg·g^−1^) represents the theoretical saturation adsorption capacity, and *K*_L_ represents the Langmuir adsorption coefficient.

Freundlich summed up a set of adsorption isothermal model equations as follows:

(5) $$\begin{array}{c} q_{e}=k_{F}C_{e}^{1/n} \end{array}$$

where *k*_F_ is the Freundlich adsorption coefficient and *n* is the equation’s characteristic constant, which indicates the parameter of the system’s adsorption capacity and can reflect the adsorption properties of the adsorbent. Prior research has shown that when 0.1 < 1/*n* < 0.5 the adsorbent can readily adsorb the target material, while when 1/*n* > 2 the adsorbent has difficulty adsorbing the target material [36]. *C*_e_ and *q*_e_ have the same meanings as in Langmuir’s equation.

#### 2.4.3. Adsorption Kinetics and Internal Diffusion Model

The fixed adsorbed boric acid solution contained 200 mg·L^−1^ of boron, and the adsorbent dose was 1.0 g·L^−1^. The strain container was filled with PP-g-GMA-NMDG and boric acid solution. The continuous temperature oscillation box was set for 2 h at 25 °C and 200 r·min^−1^, with setting times of 5, 10, 20, 30, 50, 70, 90, and 120 min. The pseudo-first-order and pseudo-second-order adsorption kinetic models, as well as internal diffusion models, were used to describe the kinetic behavior of PP-g-GMA-NMDG throughout the adsorption process.

The pseudo-first-order kinetic model is

(6) $$\begin{array}{c} \ln\left(q_{e}-q_{t}\right)=\ln q_{e}-k_{1}t \end{array}$$

where *q*_t_ (mg·g^−1^) represents the adsorption amount at time *t*, *q*_e_ (mg·g^−1^) represents the adsorption amount at the equilibrium time of adsorption, and *k*_1_ is the rate constant of the pseudo-first-order kinetic model.

The pseudo-second-order kinetic model is

(7) $$\begin{array}{c} \frac{1}{q_{e}-q_{t}}=\frac{1}{q_{e}}+k_{2}t \end{array}$$

where *q*_t_ (mg·g^−1^) is the adsorption amount at time *t*, *q*_e_ (mg·g^−1^) is the adsorption amount at the equilibrium time of adsorption, and *k*_2_ is the pseudo-second-order kinetic model’s rate constant.

The model of intra-particle diffusion is

(8) $$\begin{array}{c} q_{t}=k_{d}t^{1/2}+c \end{array}$$

where *q_t_* (mg·g^−1^) is the quantity of adsorption at time *t* and *k*_d_ (mg·g^−1^·min^−1/2^) is the diffusion constant inside the particle. When the vertical coordinate is *q_t_*, the horizontal coordinate is *t*^1/2^, and the intercept (*c* = 0) is through the origin, implying that diffusion inside the particle is the rate-limiting stage in controlling the adsorption process. Because the linear equation’s intercept (*c* ≠ 0) does not pass through the origin, internal diffusion is not the main cause of the speed limit, and the adsorption process may be influenced by additional adsorption stages.

#### 2.4.4. Thermodynamics of Adsorption

To examine the thermodynamic behavior of PP-g-GMA-NMDG during adsorption at temperatures of 25 °C, 35 °C, and 45 °C, boric acid solutions with boron concentrations of 200–400 mg·L^−1^ were used. The dosage of PP-g-GMA-NMDG was 1 g·L^−1^, and the constant temperature oscillation chamber was adjusted to 25 °C and 200 r·min^−1^ for 2 h.

The thermodynamic equation can determine the free energy change ∆*G* (kJ·mol^−1^), enthalpy change ∆*H* (kJ·mol^−1^), and entropy change ∆*S* (J·mol^−1^·K^−1^):

(9) $$\begin{array}{c} ∆G=-RT\ln K \end{array}$$

(10) $$\begin{array}{c} ∆G=∆H-T∆S \end{array}$$

(11) $$\begin{array}{c} \ln K=\frac{-∆H}{RT}+\frac{∆S}{R} \end{array}$$

where *K* is the equilibrium constant, which in Langmuir’s equation may be substituted by *k*_L_. Linear fitting is accomplished using ln*K* and 1/*T*, and the enthalpy change ∆*H* and entropy change ∆*S* can be estimated using the slope and intercept. The change in free energy can then be estimated using Equation (10).

#### 2.4.5. Selectivity in Adsorption

The binary system included B/Na^+^, B/Ca^2+^, and B/Mg^2+^, while the multivariate system consisted of B/Na^+^, Ca^2+^, and Mg^2+^, with each element at a concentration of 200 mg·L^−1^. The adsorbent was added at a concentration of 1.0 g·L^−1^. During 2 h, the constant temperature oscillation chamber was adjusted at 25 °C and 200 r·min^−1^. The effects of common cations on boron adsorbent selectivity in salt lake brine were examined.

#### 2.4.6. Stability

First, 1.0 g·L^−1^ of PP-g-GMA-NMDG was weighed and added to a boric acid solution containing 200 mg·L^−1^ boron, and a constant temperature oscillation chamber at 25 °C and 200 r·min^−1^ was established for 2 h. Following each adsorption, 1.0 mol·L^−1^ sodium hydroxide solution was used as an eluent for desorption twice for 40 min each time and the adsorbent was washed with water to neutralize it, after which the eluted adsorbent was dried in a vacuum drying oven and the process was repeated for the next adsorption.

## 3. Results and Discussion

### 3.1. Modification of UV Grafting Conditions

#### 3.1.1. Concentration of GMA

**Figure 3:**
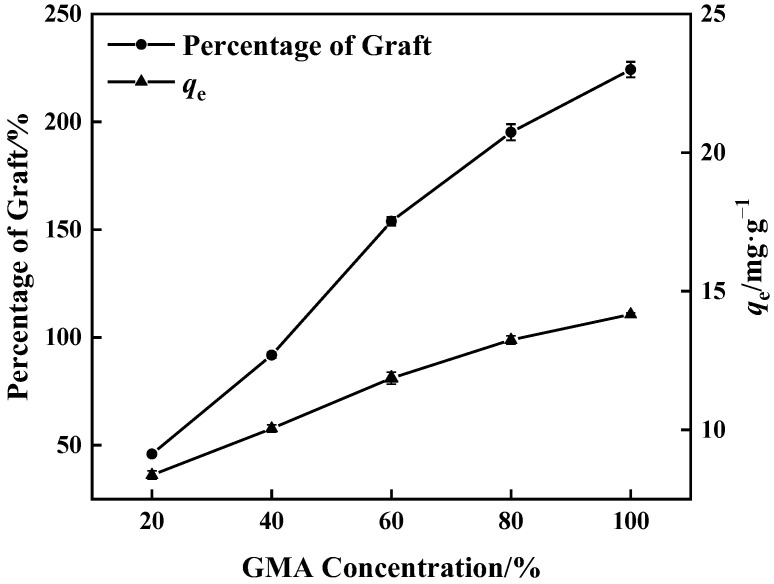
Effect of GMA concentration on percentage of graft and adsorption amount of corresponding adsorbent (0.1 g BP and 10 min grafting time).

Figure 3 shows that when GMA concentration increased, grafting of GMA on the surface of PP melt-blown fiber became more adequate and the percentage of graft increased. After the reaction of PP-g-GMA with NMDG, the percentage of grafting increased, as did the adsorption amount. The highest percentage of graft was 224%, and the corresponding maximum adsorption quantity of PP-g-GMA-NMDG was 14.17 mg·g^−1^. As the concentration approaches 60%, the growing rate of the percentage of graft falls because GMA homopolymer synthesis rises, inhibiting the occurrence of the grafting reaction [37].

#### 3.1.2. Dosage of BP

**Figure 4:**
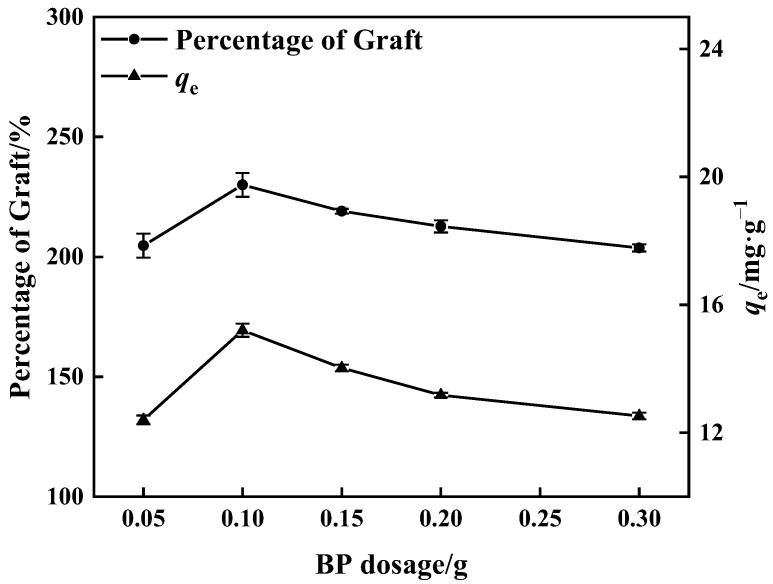
Influence of BP dosage on percentage of grafting and adsorption amount of corresponding adsorbent (100% GMA concentration and 10 min grafting time).

Figure 4 shows that when the BP dose was increased, the proportion of graft grew and ultimately declined. The greatest percentage of graft reached 230% when the BP dose was 0.10 g, and the maximum adsorption quantity of matching adsorbent was 15.20 mg·g^−1^. The excited photo-initiator molecule captures the hydrogen atom from the active monomer, low molecular prepolymer, and other hydrogen atom donors and converts it into an active free radical to commence the polymerization process. More free radicals are formed on the surface of PP melt-blown fibers when the BP dosage is increased; these polymerize with the monomers and enhance the proportion of grafting. When the dose of BP was more than 0.10 g, the high concentration of BP resulted in excess free radical production. On the contrary, the phenomenon of monomer homo-polymerization was accelerated [38,39], and the kinetic chain length was reduced, which influenced the occurrence of monomer grafting reactions and resulted in a decrease in the percentage of grafting.

#### 3.1.3. Time Required for Grafting

**Figure 5:**
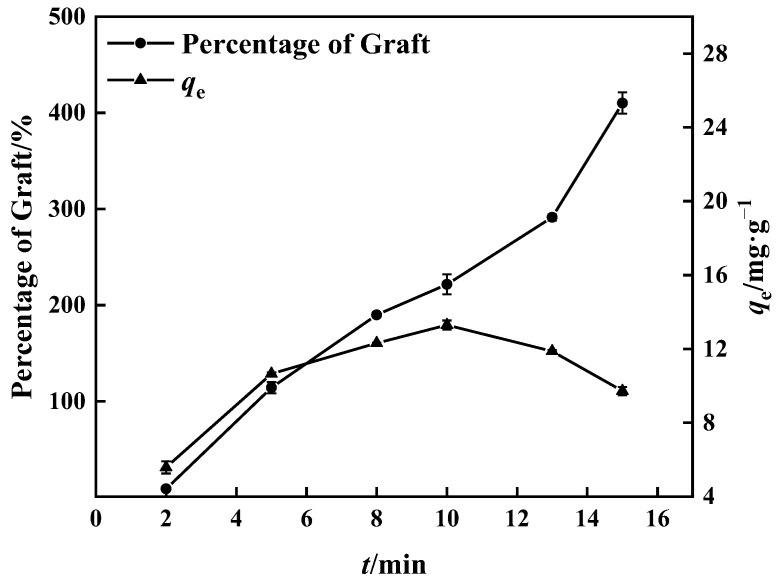
Effect of grafting time on grafting percentage and adsorption amount of corresponding adsorbent (100% GMA concentration and 0.10 g BP).

The percentage of grafting increased with time, as seen in Figure 5, because the grafting reaction was adequate owing to the rise in free radicals with time [40]. Compared to the boron adsorption test, the percentage of grafting after 15 min was greater than at 10 min, while the boron adsorption quantity was inversely related, reaching 14.00 mg·g^−1^. During the experiment, a hard and non-fluffy graft product (PP’-g-GMA) appeared in the center of the UV-grafted PP melt-blown fiber, which contrasted with the normal grafted PP melt-blown fiber around it. PP-g-GMA and PP’-g-GMA were prepared as adsorbents under the same conditions; the same mass of PP-g-GMA and PP’-g-GMA was dispersed in aqueous 1,4-Dioxane solution (*v*/*v* = 4:1), and the same amount of substance NMDG was added to it. The mixture was heated to 70 °C with mechanical stirring; the reaction lasted 8 h, and the adsorption quantity of PP’-g-GMA-NMDG dropped by nearly 50%.

**Figure 6:**
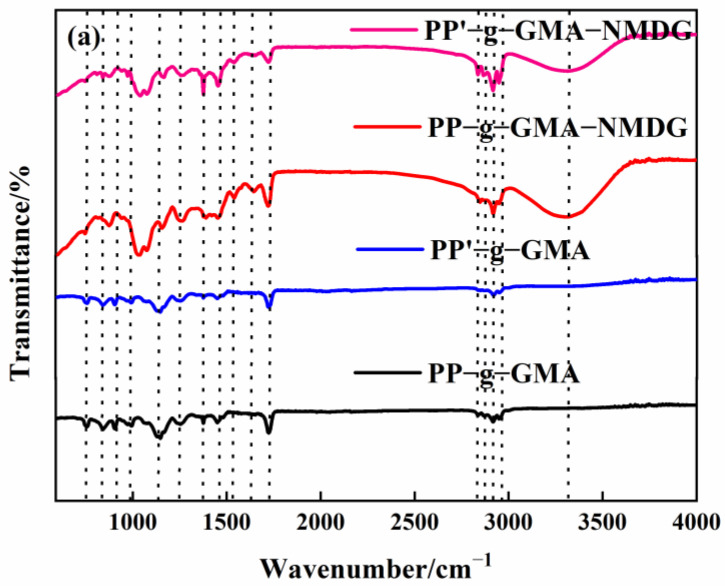

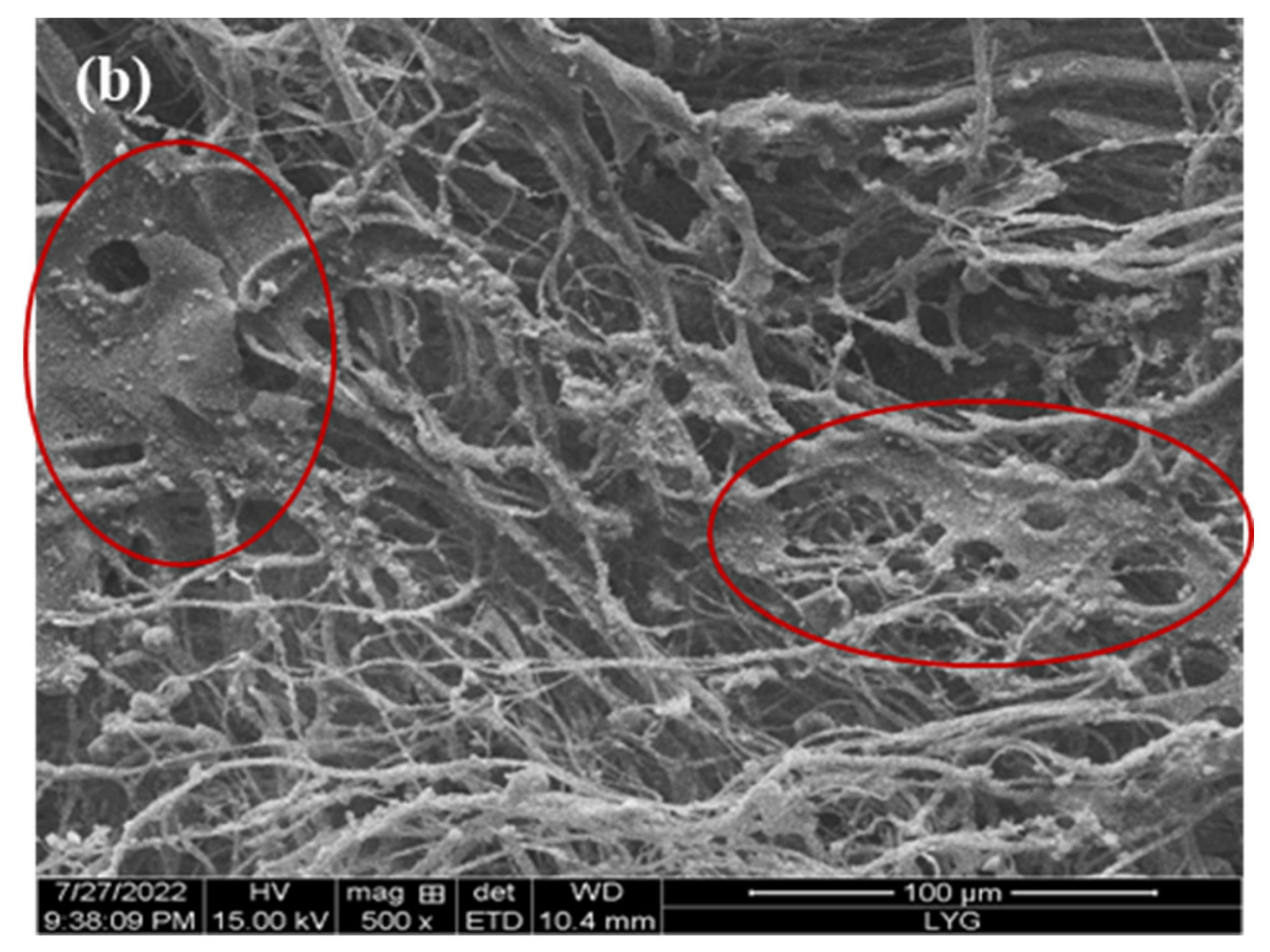
(**a**) Infrared spectrogram of PP’-g-GMA and PP’-g-GMA-NMDG; (**b**) scanning electron microscope image of PP’-g-GMA.

The internal crosslinking effect of PP melt-blown fiber following normal grafting strengthens as the grafting rate increases, though a few holes are preserved. Figure 6b shows the section where specific chemicals were applied to the interstitial area of the PP melt-blown fiber, resulting in smaller holes; the unmarked part is a regularly grafted part where GMA is connected to the PP melt-blown fiber in granular form. In comparison to PP-g-GMA and PP-g-GMA-NMDG, no new material was produced, as indicated in Figure 6a. It is likely that the GMA concentration was too high and the UV grafting duration was longer than 10 min, causing the ambient temperature to rise and resulting in acceleration of the homo-polymerization process of GMA. When too many homopolymer products are formed, this aggravates the crosslinking of PP melt-blown fiber, interferes with the grafting process, and causes the pores of PP-g-GMA to become extremely tiny. The effective contact area of PP-g-GMA then decreases in the following reaction with meglumine, which is not favorable to the reaction with meglumine, resulting in a poor adsorption effect of the product PP-g-GMA-NMDG. Nevertheless, because the homopolymer grafted onto PP melt-blown fiber is difficult to clean, the grafting rate determined by weight is very high, resulting in the grafting rate not matching the adsorption quantity.

### 3.2. Characterization

#### 3.2.1. FTIR

**Figure 7:**
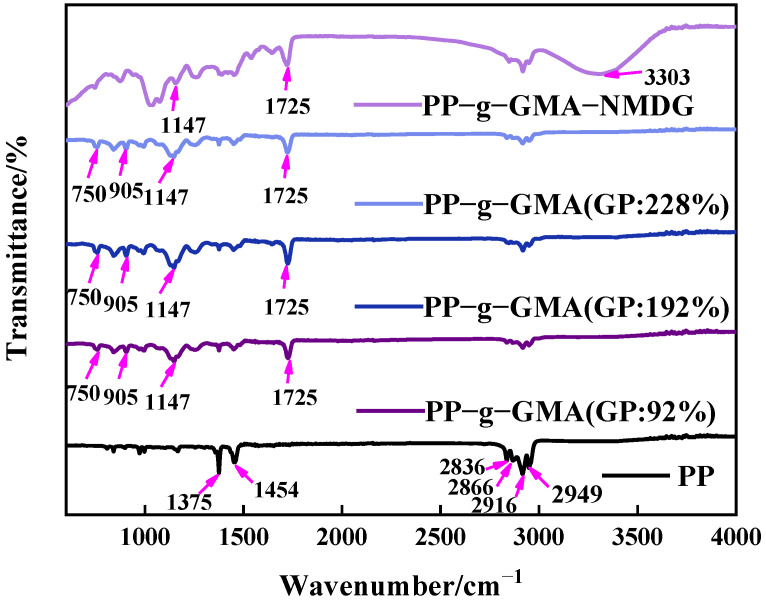
Infrared spectrum of grafted PP-g-GMA with grafting percentage of 0% to 230% and PP-g-GMA-NMDG.

PP melt-blown fiber, PP-g-GMA, and PP-g-GMA-NMDG were studied in the 500–4000 cm^−1^ range, with the findings shown in Figure 7. The evident characteristic absorption peaks of PP melt-blown fiber can be seen in Figure 6 at about 1375 cm^−1^, 1454 cm^−1^, 2836 cm^−1^, 2866 cm^−1^, 2916 cm^−1^, and 2949 cm^−1^, which are induced by the skeletal vibration of saturated alkane C-H bond [41]. There is a characteristic epoxy group absorption peak at 750 cm^−1^ and 905 cm^−1^, a characteristic absorption peak of C-O-C at 1147 cm^−1^, and a characteristic absorption peak of C=O at 1725 cm^−1^ for PP-g-GMA (GP: 46–230%), all of which demonstrate the successful grafting of GMA onto PP melt-blown fiber [42]. The distinctive absorption peak of epoxy at 750 cm^−1^ and 905 cm^−1^ vanishes, and a prominent broad peak develops at about 3303 cm^−1^, which is the characteristic absorption peak of -OH, as seen in Figure 7. These results show that GMA effectively interacts with NMDG through ring-opening and that the response is adequate.

#### 3.2.2. XRD

**Figure 8:**
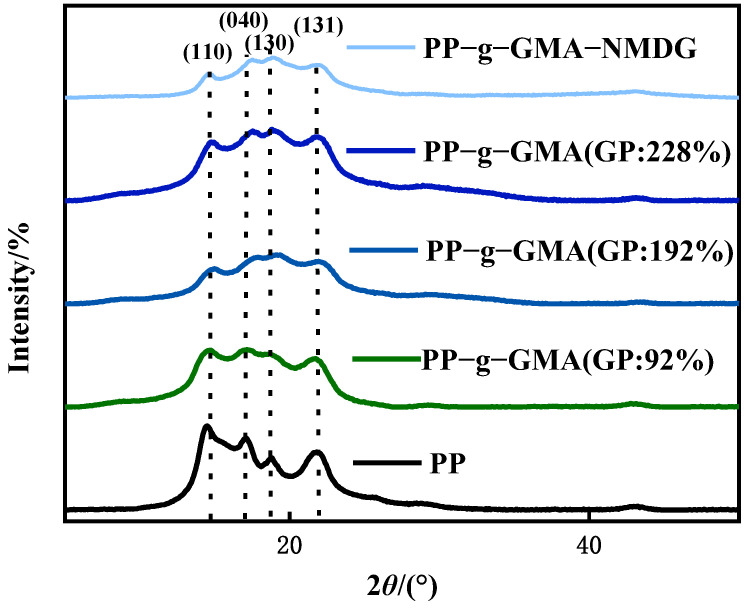
XRD patterns of PP melt-blown fiber, PP-g-GMA, and PP-g-GMA-NMDG.

Figure 8 shows the XRD patterns of PP melt-blown fiber and PP-g-GMA. The typical peaks of PP melt-blown fiber were found at 2*θ* of 14°, 17°, 18°, and 21°, which is consistent with the literature [43], and the crystal forms were (110), (040), (130), and (131), respectively. The crystal form of PP melt-blown fiber does not changed as the grafting rate increases, and the grafting of GMA has no influence on the crystal form of PP melt-blown fiber. Figure 8 shows the XRD patterns of PP melt-blown fiber and PP-g-GMA-NMDG. The peak value of PP-g-GMA-NMDG is found to be almost comparable with the 2*θ* value of PP melt-blown fiber, and PP-g-GMA-NMDG likewise retains the crystal shape of PP melt-blown fiber. The XRD pattern of the whole adsorbent production is shown in Figure 8; the fundamental crystal form of PP melt-blown fiber is not damaged throughout this procedure.

#### 3.2.3. TGA

**Figure 9:**
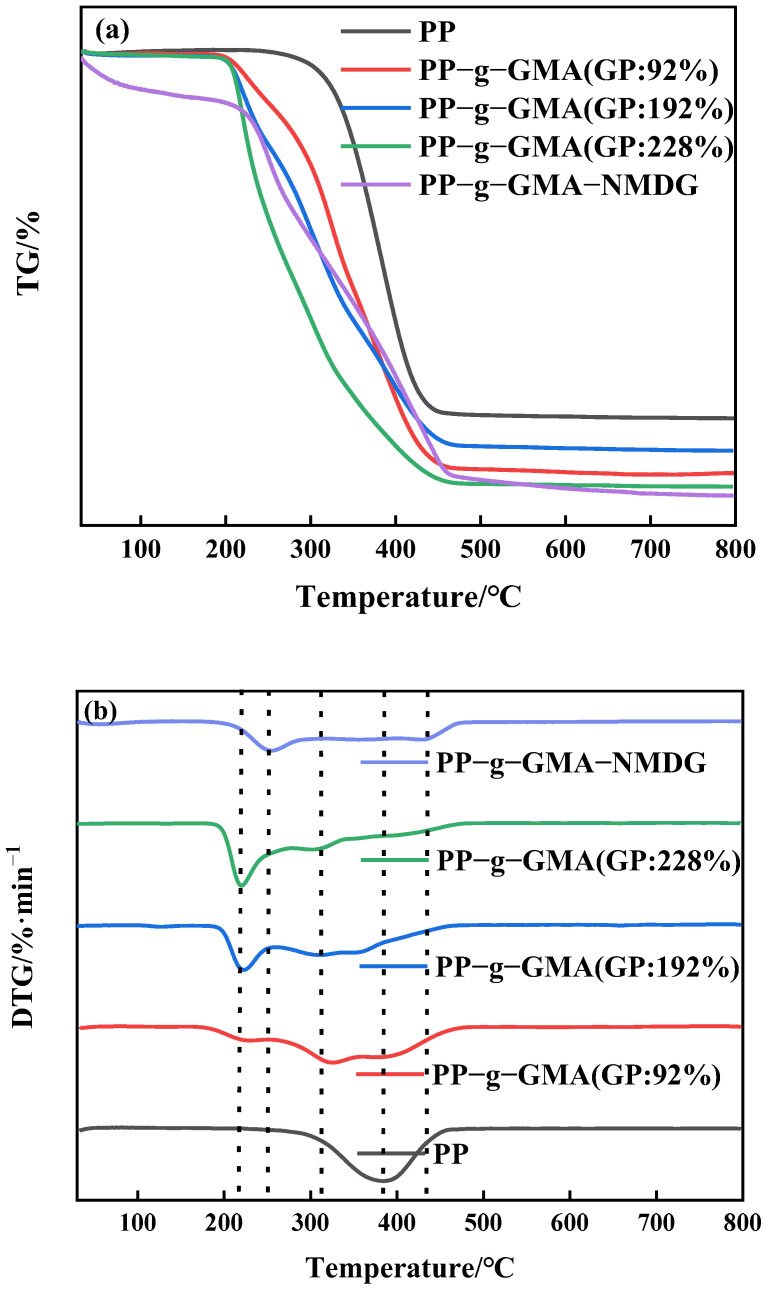
(**a**) TGA pattern of PP-g-GMA with different graft rates and PP-g-GMA-NMDG; (**b**) DTG pattern of PP-g-GMA with different graft rates and PP-g-GMA-NMDG.

The thermal stability of PP melt-blown fiber and PP-g-GMA was studied using thermogravimetric analysis in a nitrogen environment. According to Figure 9a, the PP melt-blown fiber exhibits only one degradation phase, with the greatest degradation rate occurring at about 380 °C. Figure 9b depicts the two degradation stages of PP-g-GMA. There is an additional maximum deterioration rate temperature at 220 °C in addition to the degradation temperature of the original PP melt-blown fiber. The degradation mass per minute of PP-g-GMA increases as the grafting rate increases, which is because after GMA grafting the degradation temperature of PP-g-GMA is shifted forward compared to the degradation temperature of PP melt-blown fiber; the higher the grafting rate, the more GMA content, and the degradable mass per minute of PP-g-GMA increases at around 220 °C. Figure 9a demonstrates that as the proportion of graft increases, so does the bulk of leftover residue. As shown in Figure 9b, the degradation temperature of the first stage of PP-g-GMA-NMDG is lower than that of PP-g-GMA a, indicating that PP-g-GMA successfully experienced an amination ring-opening reaction with NMDG that successfully attached the target group to the surface of the PP melt-blown fiber and changed the thermal stability performance of the PP-g-GMA. Moreover, as shown in Figure 9a, the residue decreases with the preparation of PP-g-GMA-NMDG, suggesting that there are more and more organic materials in the PP melt-blown fiber, which is compatible with the preparation process assumption.

#### 3.2.4. Contact Angle

**Figure 10:**
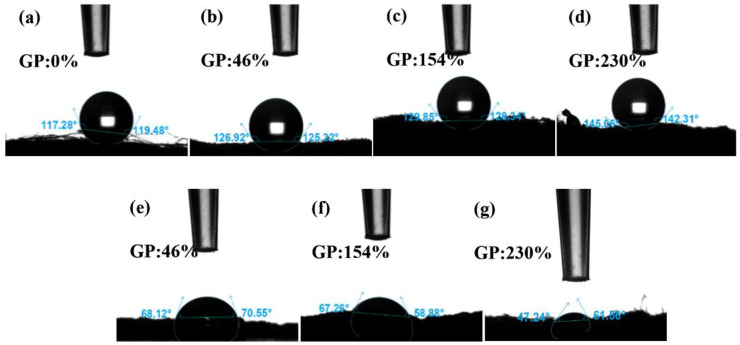
(**a**) Contact angle of PP melt-blown fiber; (**b**–**d**) GP:46–230% of PP-g-GMA contact angle; (**e**–**g**) Contact angle of PP-g-GMA-NMDG (GP of GMA: 46–230%).

The solid surface is hydrophilic if the contact angle *θ* < 90°, which means that the liquid can easily wet the solid; the smaller the angle, the greater the wettability. If *θ* > 90°, the solid surface is hydrophobic, which means that liquid does not easily wet the solid and moves easily on the surface. The hydrophobicity of PP melt-blown fiber does not change after grafting GMA, as shown in Figure 10a–d; however, the contact angle rises marginally due to GMA’s lack of hydrophilic groups [44]. Figure 10e–g depicts the contact angle of PP-g-GMA-NMDG with varying grafting percentages. The contact angle range is 47° to 70°, and there is clear hydrophilicity. This is due to NMDG’s hydrophilic hydroxyl group [45]. The hydrophilicity rises somewhat when the GMA fraction of the graft increases. This demonstrates that the surface of the PP melt-blown fiber has been effectively coupled with NMDG, considerably enhancing the hydrophilicity of the PP melt-blown fiber, and that the PP-g-GMA-NMDG was successfully manufactured.

#### 3.2.5. SEM

**Figure 11:**
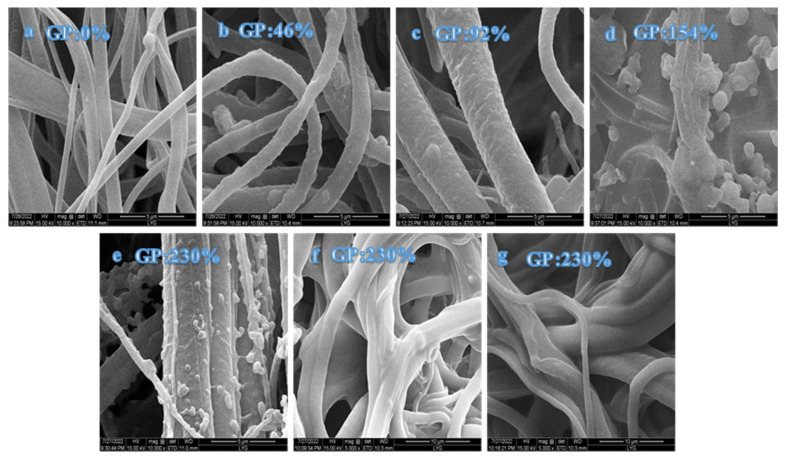
(**a**) SEM diagram of PP melt-blown fiber; (**b**–**e**) SEM diagrams of grafted PP-g-GMA with grafting rates of 46–230%; (**f**,**g**) SEM diagram of PP-g-GMA-NMDG.

SEM was used to analyze the surface morphologies and structures of PP melt-blown fiber, PP-g-GMA, and PP-g-GMA-NMDG. The SEM pictures of PP melt-blown fiber in Figure 11 demonstrate that the surface of the PP melt-blown fiber is smooth and flat, forming a fluffy and porous structure. Figure 11b–e shows scanning electron microscopy pictures of various grafting percentages (46–230%), with increasing proportion of grafting. The surface of the PP melt-blown fiber becomes rough and uneven, and particles are stuck to the PP melt-blown fiber. Compared with the previous Figure 6b, it can be seen that the normally-grafted GMA is adhered to the PP melt-blown fibers in the form of particles, as opposed to the cross-linking phenomenon in Figure 6b. These particles increase dramatically in number as the fraction of grafting increases. When the GP is 154–230%, the surface of the PP melt-blown fiber becomes smooth rather than rough, and particles are stuck to the surface of the PP melt-blown fiber. This might be because GMA generates particles that adhere to the surface of the PP melt-blown fiber. The particles on the surface of the melt-blown fiber vanish, as seen in Figure 11f,g. According to the characterization data, the ring-opening amination reaction was effectively carried out by NMDG and GMA, causing the surface of the PP melt-blown fiber to become smooth.

### 3.3. Adsorption Mechanism of PP-g-GMA-NMDG

#### 3.3.1. Relationship between pH and Adsorption Capacity

**Figure 12:**
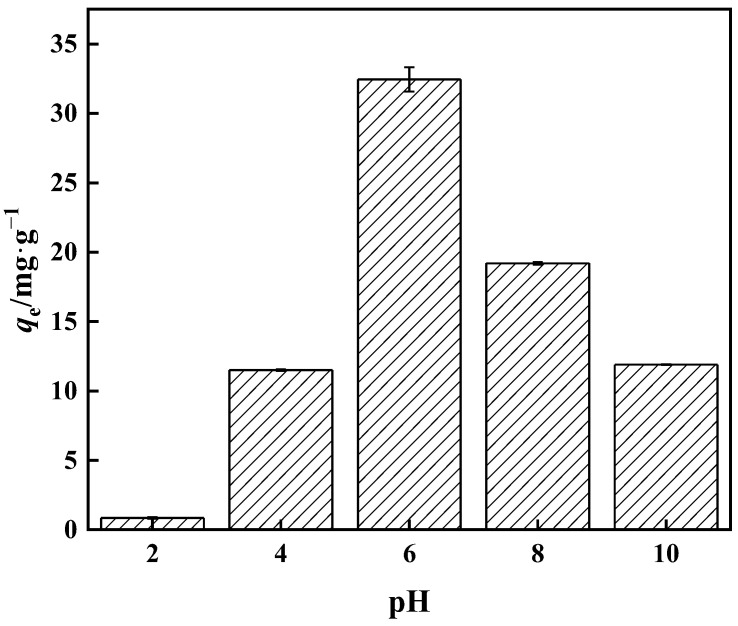
Effect of pH on PP-g-GMA-NMDG adsorption capacity.

When the overall concentration is less than 25 mmol·L^−1^, the aqueous solution consists mostly of H_3_BO_3_ and B(OH)_4_^−^, with no additional anions present. The majority of the boron in water resides as H_3_BO_3_ at low pH. The amount of B(OH)_4_^−^ in boric acid aqueous solution increases as the pH rises. The proportion of B(OH)_4_^−^ reaches 80% when the pH is 9.4 [46]. Among the many forms of boric acid, only B(OH)_4_^−^ can swiftly build a stable five-membered ring complex with an *o*-dihydroxyl group. The adsorption capacity of the adsorbent with respect to borate progressively rises with pH increase, as seen in Figure 12, as the higher the pH means a greater the amount of H^+^ in the solution. The H^+^ and borate ions compete for the adsorption sites on PP-g-GMA-NMDG, reducing the target ion’s adsorption capability. The adsorption amount reaches a peak at pH 6, where the adsorption amount is 32.00 mg·g^−1^. As the pH continues to rise, the adsorption capacity decreases. This is because when the pH rises the concentrations of OH^−^ and B(OH)_4_^−^ rise as well, and the electrostatic repulsion of excess OH^−^ and B(OH)_4_^−^ in the aqueous solution increases, resulting in a reduction in adsorption capacity.

#### 3.3.2. Adsorption Isotherm

**Figure 13:**
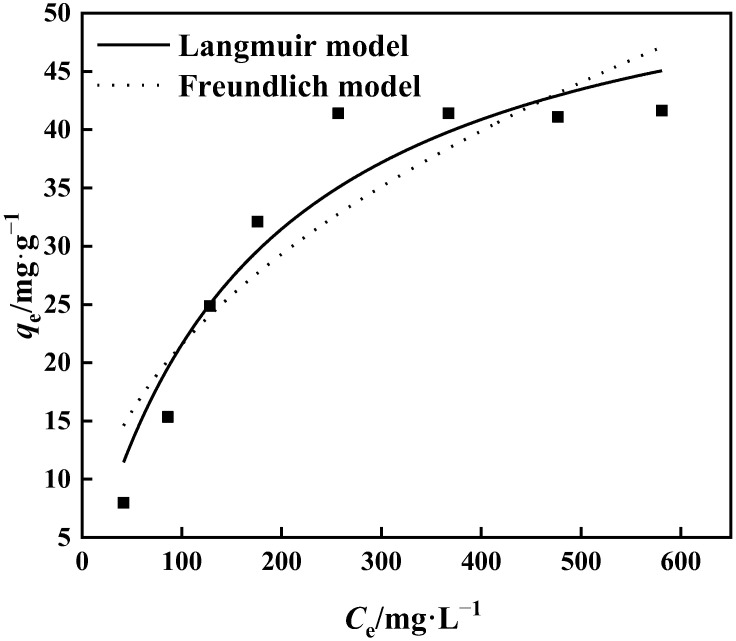
Langmuir and Freundlich fitting of adsorption isotherms.

The connection between the initial concentration of the adsorption solution and the equilibrium adsorption capacity is shown in Figure 13. It can be seen that the adsorption capacity of PP-g-GMA-NMDG rises as the starting concentration increases. The adsorption capacity of PP-g-GMA-NMDG approaches adsorption saturation at a concentration of 300 mg·L^−1^, and the highest saturated adsorption capacity is 41.65 mg·g^−1^. The isothermal behavior of the adsorption process was analyzed using the Langmuir model and the Freundlich model at 25 °C to evaluate the isothermal adsorption behavior of PP-g-GMA-NMDG. Figure 13 and Table 1 illustrate the findings. The *R*^2^ value for the Langmuir model is 0.924, and the maximal theoretical adsorption capacity is 58.26 mg·g^−1^. The Freundlich model calculates 1/*n*, and the result is less than 0.5, as shown in Table 1. As a result, PP-g-GMA-NMDG readily absorbs target ions. In conclusion, the isothermal adsorption process of the adsorbent is more in accordance with the Langmuir model at 25 °C and starting concentration range of 50–600 mg·L^−1^, suggesting that the adsorption process is suited for monolayer adsorption and is evenly adsorbed on solid surfaces [47].

#### 3.3.3. Kinetics of Adsorption and Internal Diffusion Model

**Figure 14:**
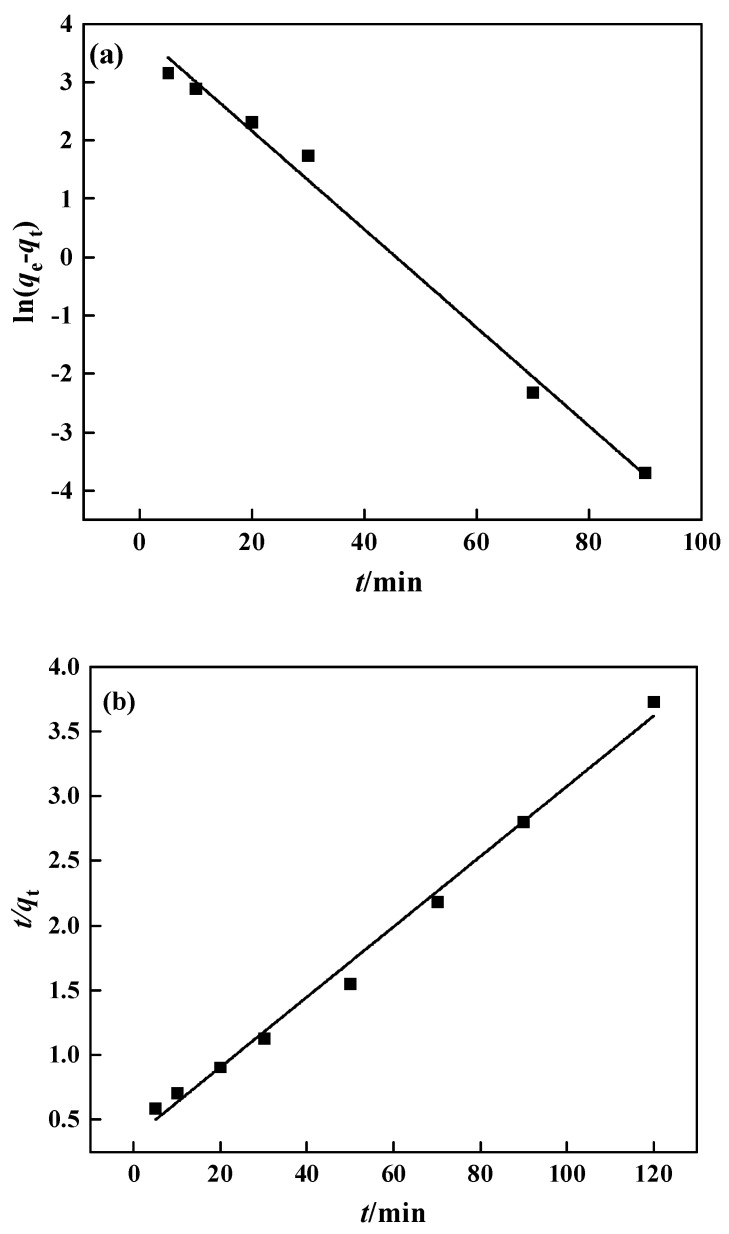
PP-g-GMA-NMDG kinetic fitting: (**a**) pseudo-first-order model and (**b**) pseudo-second-order model.

The adsorption rate is relatively fast in the first 20 min and gradually slows down after that, reaching adsorption equilibrium about 50 min later. In this experiment, the pseudo-first-order and pseudo-second-order models were chosen for fitting, with the results displayed in Figure 14 and Table 2. The pseudo-second-order kinetic equation fits the experimental data quite well, with a correlation of 0.993, and the theoretical maximum adsorption capacity was 36.87 mg·g^−1^, which is close to the observed maximum adsorption capacity of 31.15 mg·g^−1^. The pseudo-second-order kinetic equation better describes the PP-g-GMA-NMDG adsorption process, indicating that chemical adsorption is the key limiting factor on the adsorption rate in this process [48].

**Figure 15:**
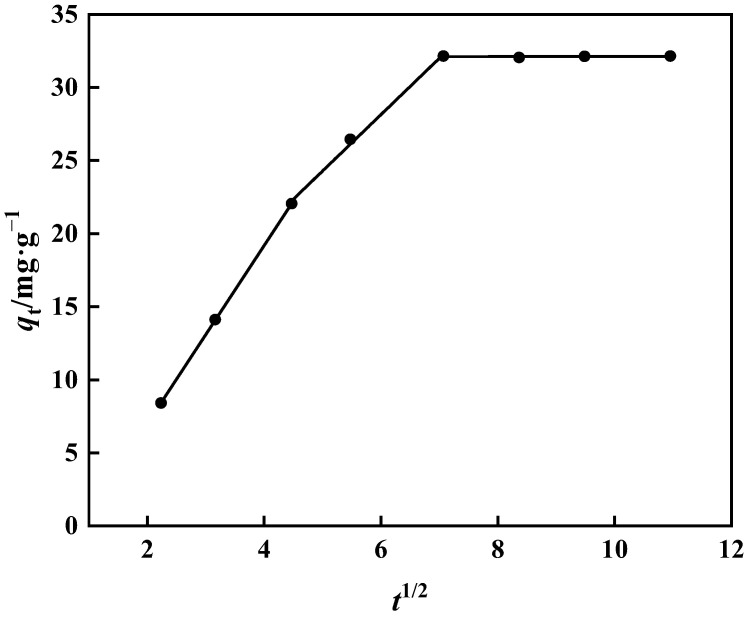
Internal diffusion model of PP-g-GMA-NMDG adsorption process.

The fitting of the internal diffusion model does not travel via the origin, as seen in Figure 15 and Table 3. As a result, it is clear that extramembrane and intramembrane diffusion play a significant role in the adsorption of boric acid by PP-g-GMA-NMDG. Table 3 shows the slope: *k*_d1_ > *k*_d2_ > *k*_d3_. Borate binds to PP-g-GMA-NMDG in two stages. As borate ions reach the adsorption site on the adsorbent’s surface, it progressively transforms into intra-particle diffusion of PP-g-GMA-NMDG, and the adsorption rate becomes proportional to the thickness of the boundary layer. Adsorption steadily slows as the thickness of the boundary layer increases, eventually reaching adsorption equilibrium [49].

#### 3.3.4. Adsorption Thermodynamics

**Figure 16:**
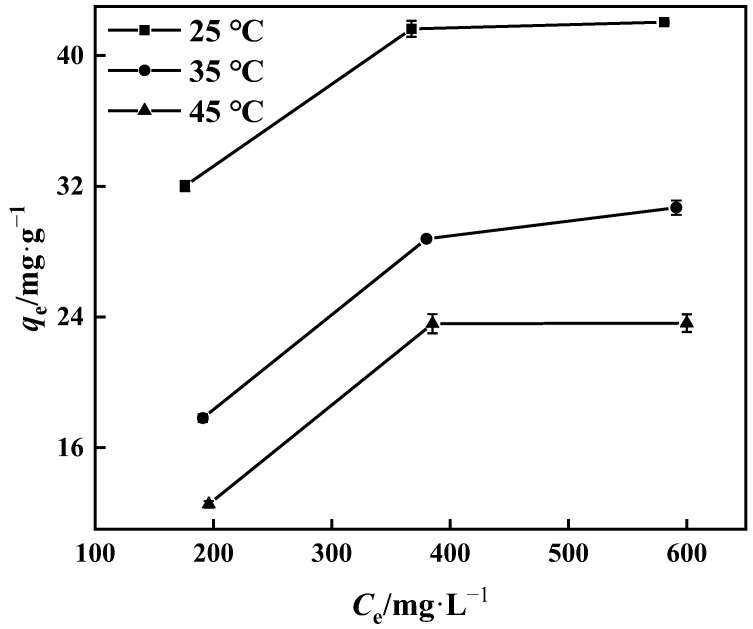
The influence of temperature on the adsorption capacity of PP-g-GMA-NMDG.

**Figure 17:**
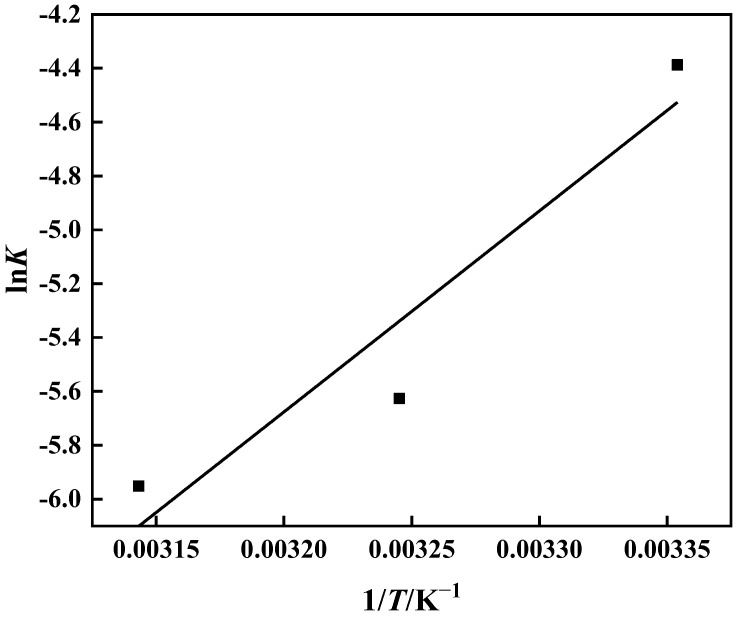
Logarithm of the equilibrium constant ln*K* and reciprocal temperature 1/*T*.

The thermodynamic behavior of the PP-g-GMA-NMDG adsorption process was studied at three different temperatures (25 °C, 35 °C, and 45 °C). Figure 16 shows that the adsorption of PP-g-GMA-NMDG decreases with increasing temperature, indicating that the adsorption process is exothermic. The adsorption isotherm simulation shows that the process conforms to the Langmuir model, and the adsorption equilibrium constant *K* of the process can be determined from the Langmuir model at these three temperatures. The adsorption process’s ∆*H*, ∆*S*, and ∆*G* are determined from the slope and intercept derived from the appropriate equation in Figure 17 [50]; Table 4 shows the outcomes. The computed ∆*H* value is negative, suggesting that the process is exothermic, in agreement with the previous conjecture. The ∆*G* values at the three temperatures are all less than zero, showing that the process is spontaneous. In addition, the ∆*S* value is larger than zero. It is possible that the surface structure of PP-g-GMA-NMDG is altered to some extent, increasing the chaos of the adsorption system.

#### 3.3.5. Adsorption Selectivity

**Figure 18:**
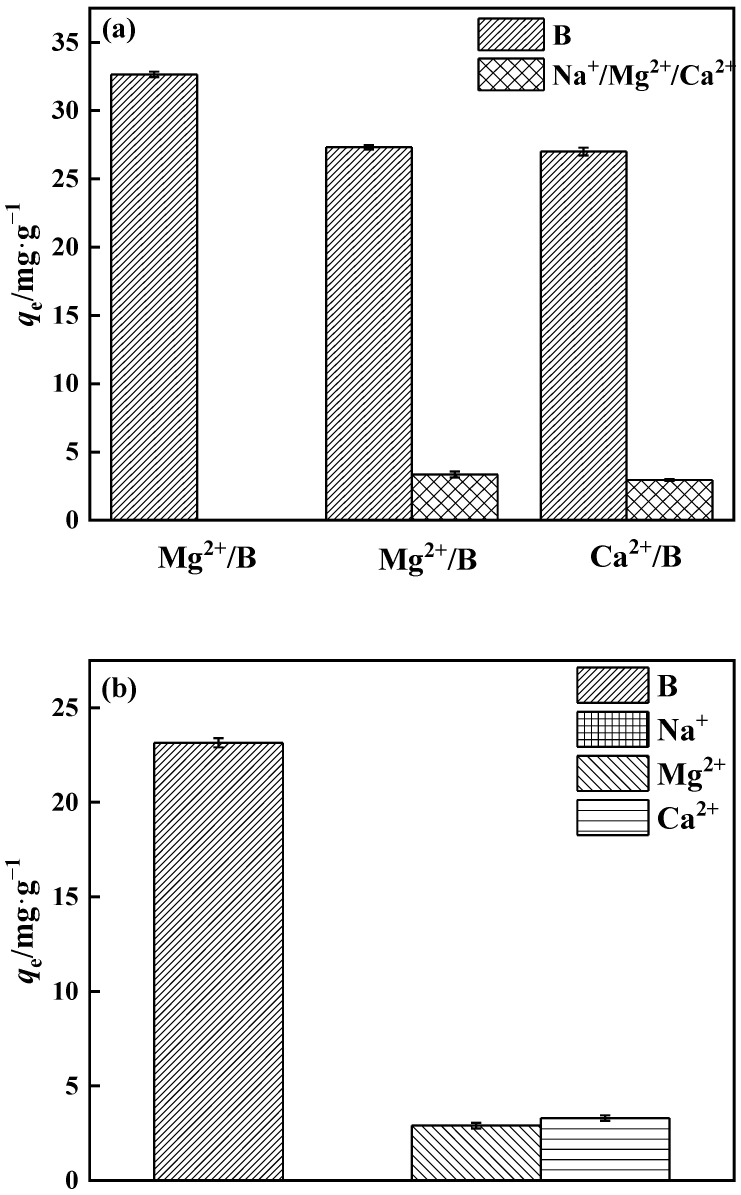
PP-g-GMA-NMDG selectivity in (**a**) binary and (**b**) multiple systems.

The quantities of Na^+^, Mg^2+^, and Ca^2+^ in salt lake brine are relatively high. As a consequence, the selectivity of PP-g-GMA-NMDG for borate adsorption in binary and multicomponent systems was investigated (Figure 18a). The binary system of PP-g-GMA-NMDG has minimal adsorption on Na^+^, Mg^2+^, and Ca^2+^, and the amount of boron adsorption decreases in the Mg^2+^ and Ca^2+^ systems. The polyhydroxy adsorption sites on PP-g-GMA-NMDG may have electrostatic affinity for Ca^2+^ and Mg^2+^, occupying just a small part of the adsorption sites. Polyhydroxy molecules have a higher proclivity to bond with boric acid because they include many o-dihydroxy groups. As shown in Figure 18b, the boron adsorption capacity of PP-g-GMA-NMDG decreased by approximately 29% in the multicomponent system, while the adsorbent’s overall selectivity for borate ions remained unchanged. While certain metal ions can have an effect on adsorption capacity, in general this effect is minimal. It is probable that PP-g-GMA-NMDG has a better selectivity for borate ions.

#### 3.3.6. Reusability

**Figure 19:**
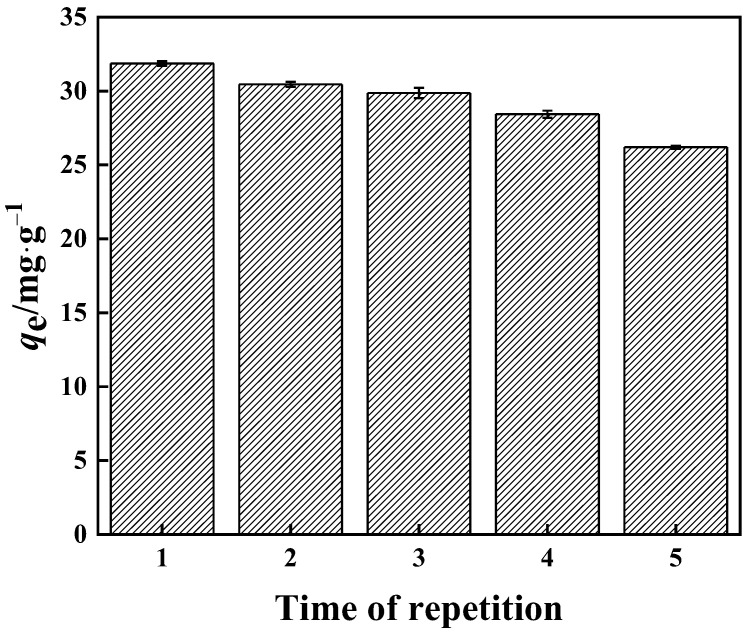
Performance of PP-g-GMA-NMDG reuse.

In this work, the reusability of the PP-g-GMA-NMDG adsorbent was investigated. The finished product can be seen in Figure 19. When the adsorbent was used for the second time, its adsorption capacity decreased from 31.00 mg·g^−1^ to 30.50 mg·g^−1^, and its utilization performance decreased by around 1.6%. When the adsorbent was used for the fourth time, its adsorption capacity dropped to roughly 28.00 mg·g^−1^ and its utilization performance dropped by nearly 10%. The adsorption capacity was roughly 26.20 mg·g^−1^ after the fifth usage, and the utilization performance was nearly 15% lower. PP-g-GMA-NMDG retains approximately 85% of its adsorption performance after five cycles of adsorption, and the adsorbent’s overall reuse performance remains good.

In this study, the highest adsorption capacity of PP-g-GMA-NMDG was 41.65 mg·g^−1^. According to current research on boron adsorbent materials, shown in Table 5, the adsorption capacity of the adsorbent in this experiment is more desirable than that of other adsorbents. The advantages of this adsorbent are in both the adsorption amount and its selectivity and reproducibility. The adsorbent substrate material is PP melt-blown fiber, which is stable, inexpensive, and easy to recover. The combined advantages of this adsorbent may be ideal for the practical application of boron extraction from salt lake brine.

## 4. Conclusions

In this work, a boron adsorbent was produced utilizing a simple green synthesis procedure and UV grafting modification technology. To optimize the UV grafting conditions, a single factor experiment was employed. The GMA concentration was 100%, the BP dose was 0.1 g, and the grafting duration was 10 min, which might have minimized byproduct generation in the process. To further understand the stability of the material, the substance was characterized. First, using FT-IR, we successfully connected our intended groups to the surface of PP melt-blown fiber. TGA and XRD examination revealed that the PP melt-blown fibers were thermally stable and retained their original crystalline form well. The contact angle measurement greatly increased the hydrophilicity of PP-g-GMA-NMDG. Finally, SEM images were used to examine the adsorbent’s morphology. The adsorbent is hydrophilic and stable, with both groups capable of boron adsorption while retaining the fundamental properties of PP melt-blown fibers. To further understand the adsorption process, the adsorption mechanism of PP-g-GMA-NMDG was studied. The adsorption process of this adsorbent was more consistent with the pseudo-second-order model and the Langmuir model, and the actual maximum adsorption capacity of PP-g-GMA-NMDG was 41.65 mg·g^−1^. Furthermore, the adsorbent was made of PP melt-blown fiber, which is easy to separate and recover, has good selectivity, and has the potential to be used in boron extraction.

## Figures and Tables

**Table 1:** Fitting coefficients of the Langmuir and Freundlich models.

Langmuir			Freundlich		
*k*_L_	*q*_m_ (mg·g^−1^)	*R* ^2^	*k*_F_	1/*n*	*R* ^2^
0.006	58.26	0.924	2.782	0.444	0.836

**Table 2:** Parameters of the PP-g-GMA-NMDG dynamic model.

*q*_e_ (mg·g^−1^)	Pseudo-First-Order Model			Pseudo-Second-Order Model		
	*q*_m_ (mg·g^−1^)	*k*_1_	*R* ^2^	*q*_m_ (mg·g^−1^)	*k*_2_	*R* ^2^
31.15	46.81	0.084	0.992	36.87	0.002	0.993

**Table 3:** Internal diffusion model parameters of PP-g-GMA-NMDG adsorption process.

*k*_d1_ (mg·g^−1^·min^−1/2^)	*R* ^2^	*k*_d2_ (mg·g^−1^·min^−1/2^)	*R* ^2^	*k*_d3_ (mg·g^−1^·min^−1/2^)	*R* ^2^
6.095	0.999	3.858	0.997	0.006	0.041

**Table 4:** Adsorption thermodynamic characteristics of PP-g-GMA-NMDG at 25 °C, 35 °C, and 45 °C.

*T*/°C	Thermodynamic Parameter			*R* ^2^
	∆*H* (kJ·mol^−1^)	∆*S* (J·mol^−1^·K^−1^)	∆*G* (kJ·mol^−1^)	
25 °C	62.063	245.796	135.347	0.909
35 °C			137.805	
45 °C			140.263	

**Table 5:** Comparison of boron adsorption by different adsorbents.

Adsorbents	Substrate Material	*q*_e_ (mg·g^−1^)	References
Zr-NU-1008	MOFs	23.28	[51]
Hf-NU-1008		24.56	
Zr-NU-903		19.66	
Hf-NU-903		28.02	
Zr-NU-1008 *		9.83	
Hf-NU-1008 *		12.12	
CTS-MG	Chitosan	20.36	[10]
Zr-CTS	Chitosan	22.2–24.5	[52]
S-VBC-NMDG	Sulfur-based polymers prepared from sulfur and 4-vinylbenzyl chloride (VBC) revulcanization	7.186	[53]
P(GMA-co-TRIM)-EN-PG	Glycide-functionalized polymeric nanomaterials	29.22	[54]
P(GMA-CO-TRIM)-TETA-PG		23.25	
PP-g-GMA-NMDG	PP melt-blow fiber	41.65	This work

## Data Availability

All data are contained within the article.
